# Lectures on the Diagnosis and Treatment of Diseases of the Heart

**Published:** 1841-12-04

**Authors:** W. W. Gerhard


### MEDICAL EXAMINER.

DEVOTED TO MEDICINE, SURGERY, AND THE COLLATERAL SCIENCES.

No. 49.] PHILADELPHIA, SATURDAY, DECEMBER 4, 1841. [Vol. IV.

### LECTURES ON THE DIAGNOSIS AND TREATMENT OF DISEASES OF THE HEART.

BY W. W. GERHARD, M. D.

### LECTURE XX.

#### HYPERTROPHY OF THE HEART.

The heart, like all hollow organs, is subject to thickening or hypertrophy. The disease is rather one of slow nutrition than of any active disturbance, never occurring in a very short time, and resulting either from the effects of various acute diseases which have left more or less permanent lesions after them, or from some long continued stimulant acting slowly upon the organ.

Hypertrophy is divided into three varieties, The first, or simple hypertrophy, is that in which the thickness of the muscular tissue is increased, without much alteration of the valves, or dilatation of the cavities. This variety lasts for a very long period, and in many cases seems scarcely to shorten the life of the patient, or to produce much disturbance of the functions. It is rather a favourer of diseases of other organs than a cause of death by the derangement of the action of the heart. The second variety is neither common nor very important. It is termed concentric hypertrophy: the thickness of the walls of the heart is increased, but only in the interior, so that the size of the whole organ does not appear at all or, at least, much greater than usual, while the walls are found to be much thickened if they are cut into, and the cavities proportionally diminished, the thickening taking place mainly at their expense. If this lesion is carried to a great degree, it will produce a decided impediment to the circulation; but, practically, this degree of alteration is rarely met with. The third and last variety is hypertrophy combined with dilatation, the most severe and the most intractable variety of this disease. The danger partly arises from the direct effects of the lesion, and partly from the complications which generally precede and aggravate the hypertrophy. Like the other, this variety is slow in growth, but from time to time it takes on a sudden and rapid increase from attacks of acute inflammation of its serous membrane.

The *anatomical characters* of hypertrophy vary so far as the size and conformation of the heart are concerned; but they possess several characters in common. The tissue of the heart is not increased in thickness, but it becomes harder and more resisting than the natural muscle,—in some cases nearly as firm and as difficult to cut as cartilage. The colour of the heart is redder than usual, and even in those cases in which the patient has become anemic, the redness persists for a very long period after the other tissues of the body. The shape of the heart remains nearly natural in concentric hypertrophy, but in the simple, and still more in the dilated variety, the organ becomes more rounded, and approaches to the spherical form.

*Causes.*—These vary according to the nature of the lesion. In the simple variety the cause is generally mild and slow in its action, producing a gradual increase of nutrition. Sometimes the heart is disproportionately large from original conformation, or from some unknown cause acting in early life; but in general no variety of the disease occurs until puberty, and the proportion of cases becomes greater and greater as the patient advances in life. Active muscular exertion, especially if conjoined with powerful action of the muscles of the chest, which impedes the respiration and circulation, frequent attacks of slight muscular rheumatism, nervous disorder of the heart, and acute inflammation of the organ badly cured, may all give rise to this variety. The causes of the second are unknown, but they are more frequently referrible to inflammation, or to slow rheumatic attacks, than to any other. The third variety is nearly always more or less dependent upon inflammation; sometimes it commences during the attack of endocarditis, but in most instances it is caused by the obstruction to the circulation which results indirectly from the valvular thickening occurring during the acute attack. The heart is thrown into violent action by the effort necessary to force the blood through the thickened valves, or to repel it backwards when the opening is permanently dilated. There are other cases, although less frequent, in which no acute cause can be discovered, and the hypertrophy results either from long continued muscular efforts, or from slowly acting irritation, especially gout or muscular rheumatism.

*Signs and Symptoms.* The physical signs of hypertrophy are generally quite conclusive. In the simple varieties, they belong exclusively to the lesion itself, but, when the heart is dilated, the signs are more or less mingled with those of valvular disease and of dilatation. In simple hypertrophy we have three well-defined physical signs: the first of these is the increase of the impulsion. The force of impulsion depends partly upon the quantity of muscle, and partly on its power or activity; and, as in hypertrophy, the size is necessarily increased, while the tissue of the heart becomes stronger and firmer; there is necessarily an increase in the power of impulsion, which is only lost when the energies of all the muscles decline on the near approach of death. The impulsion is not only increased in force, but in extent, the heart evidently applying itself over a larger surface, and raising itself gradually against the ribs with a heaving motion, which is totally different from the short, quick stroke of nervous or functional disturbance. In other words, the observer feels that there is a positive increase of momentum dependant upon a large mass pressing against the walls of the chest. In all the varieties of hypertrophy the increased impulsion forms one of the most characteristic signs, but it is of course less in the concentric than in the other varieties, in which the size of the organ, considered as a whole, is increased. It is greatest in some cases of hypertrophy with dilatation, in which the walls of the heart are excessively thickened. The increased force of impulsion may be readily calculated by a reference to a normal and a hypertrophied heart; in the natural state the thickness is not usually more than a third to half an inch increased at the middle of the left ventricle, which we generally take as the standard. The normal thickness of course varies, just as that of any other muscle of the body, depending upon the general development of the individual, the sex, stature, &c. Hence the heart is rather thicker in males than in females, and in those who have followed laborious employments than in persons who have led an idle, inactive life; in the well-nourished, than in those whose digestion is impaired, or diet insufficient.

The mode in which the impulse is formed is also peculiar; instead of a short decided stroke, it is heaving; that is, point after point of the heart is applied against the walls of the chest, which gives to the observer the sensation of a large massive organ, and not simply increased energy and rapidity of blow. As the total size of the heart is greater in hypertrophy with dilatation than in any other variety, the heaving motion is then of course most perceptible.

The sounds of the heart are more or less changed in hypertrophy: even in the most simple variety the sounds become less sharp, especially the second, and the first is more or less prolonged, approaching insensibly to the bellows sound. In some cases the second sound disappears entirely; in others it retains its natural distinctness; and in the third variety it may even become louder than natural; for, as the necessary effect of dilatation is to increase the loudness and sharpness of the second sound, the deadening effect of the hypertrophy is more than neutralized. In such cases we often find a prolonged and rough bellows sound, while the second is clear and distinct, but the valves must of course remain in the normal condition.

The degree of hypertrophy may also be measured by percussion, and by the prominence which is almost always produced after a time. The prominence is most readily formed in young persons whose cartilages are elastic, and yield readily to the long continued effects of the heart. Like the enlarged heart, the prominence is more or less oval in shape, and is of course much greater in those cases in which hypertrophy is conjoined with dilatation, than in the simpler varieties. The results of percussion are much more satisfactory; not only do we ascertain the actual limits of the heart, but we can, with much accuracy, ascertain if the thickening is great at the centre of the organ. In such cases the dulness of sound is replaced by complete flatness over the centre, and although the extent of dulness which is naturally observed may not be increased, it may become so much more evident, and so much greater in degree, that our diagnosis is equally sure. In cases where there is no dilatation, the degree of dulness at the centre of the heart is a much better indication than the mere extension of it.

The *sensations* felt by the patient in the chest are at times a good guide for the diagnosis. If the chest should happen to be narrow and the heart more or less compressed, the strong impulsion is proportionably much more distressing to the patient, who then complains of the violent throbbing; but a full, capacious thorax with firm ribs, is by no means so apt to feel the impulsion, and the thickening may go on to a great degree without causing much uneasiness. Besides the palpitation, there is often much suffering from dyspnœa, which arises, partly from the difficulty in expelling the blood from the heart, if the auriculo-ventricular valves should be patulous, or the semilunar valves contracted, and partly from the secondary effects of this impediment, which congests the lungs and prevents their full expansion. Pains are also complained of from time to time; these are directly dependent upon the disease, and are vague and wandering, sometimes extending down the left arm, as in other diseases of the heart, or they are secondary, and depend upon the accidental attacks of inflammation of both serous membranes of the heart, which are almost sure to recur from time to time. In many patients there is scarcely pain, but merely a vague sense of uneasiness, at times scarcely felt, at others more resembling that of a weight pressing at the region of the heart.

The *vascular system* is necessarily affected in cases of hypertrophy. The arteries are strongly distended by the powerful action of the enlarged heart, unless the aortal valves are contracted, when the pulse may become small and irregular; this, however, results from the latter cause, and not from the hypertrophy itself, which always tends to increase the firmness and fulness of the pulse. The capillary system feels the impulsion as well as the arterial, and congestions often occur at different parts of the body; hence the subjects of hypertrophy are liable to hæmorrhages, especially from the nose and the lungs, to hæmorrhoids, and to apoplexy of the brain and lungs. The hæmorrhages become less frequent as the strength of the patient declines, and the congestions connected with them become more passive in their character; but in the early periods of the disease the natural effect of a strong impulsion of the heart is nearly always perceptible upon the capillary system. The passage of the blood through it becomes obstructed, and external hæmorrhage is a natural mode of relief; but if internal, though a similar effort of nature to relieve the vessels, it is often a cause of death. The veins are very little distended in simple hypertrophy; like the rest of the vascular system, they are generally well supplied with blood in the early stages of the disease, but they do not present any marked pulsations unless the tricuspid valve should yield to the continual stretching of its fibres, when regurgitation is apt to take place, and, of course, pulsation of the veins follow. The dilatation of the valve is most apt to occur in the third variety of hypertrophy, because it must participate after a time in the general enlargement or the cavities of the heart.

